# Improvement of hybrid grouper (*Epinephelus fuscoguttatus* ♀ × *E. lanceolatus* ♂) by enzyme-digested poultry by-product: Growth performance, amino acid and peptide transport capacity, and intestinal morphology

**DOI:** 10.3389/fnut.2022.955734

**Published:** 2022-07-19

**Authors:** Xuanyi Yang, Xumin Zhao, Guanghui Wang, Xiaohui Dong, Qihui Yang, Hongyu Liu, Shuang Zhang, Beiping Tan, Shuyan Chi

**Affiliations:** ^1^Laboratory of Aquatic Nutrition and Feed, College of Fisheries, Guangdong Ocean University, Zhanjiang, China; ^2^Yichang Huatai Biological Technology Co., Ltd., Yichang, China; ^3^Aquatic Animals Precision Nutrition and High Efficiency Feed Engineering Research Center of Guangdong Province, Zhanjiang, China; ^4^Southern Marine Science and Engineering Guangdong Laboratory, Zhanjiang, China

**Keywords:** growth performance, amino acid and peptide transporters, enzyme-digested poultry by-product meal, intestinal microbiota, non-specific immunity, hybrid grouper

## Abstract

**Background:**

At present, fish meal (FM) resources are in short supply, and competition for food between humans and animals is becoming increasingly critical. Finding non-grain protein sources that can replace FM is the key to solving the rapid development of aquaculture.

**Methods:**

Seven trial diets were prepared with 0 g/kg (EP0), 30 g/kg (EP3), 60 g/kg (EP6), 90 g/kg (EP9), 120 g/kg (EP12), 150 g/kg (EP15), and 180 g/kg (EP18) of enzyme-digested poultry by-product meal (EPBM) by replacing of FM. A total of 630 hybrid groupers (*Epinephelus fuscoguttatus* ♀ × *E. lanceolatus* ♂) were equally portioned into 21 tanks. At 8:00 and 16:00 each day, groupers were fed until they were full for a cumulative period of 8 weeks.

**Results:**

The results showed that 30 g/kg of EPBM significantly increased the rates of weight gain and special growth (*P* < 0.05). Significantly higher activities of serum glutamic pyruvic transaminase, glutamic oxaloacetic transaminase, catalase, and superoxide dismutase were observed in the EP3 group (*P* < 0.05). The categories and numbers of the top 10 dominant bacteria in the phylum and genus levels were not significantly influenced by feed (*P* > 0.05). In the proximal intestine and distal intestine, there were significantly higher expressions of *SNAT3, LAAT1, CAT2*, and *CAT1* in the EP3 group compared with the EP0 group (*P* < 0.05). In the EP3 group, the expressions of *PepT1, LAAT1, B^0, +^AT*, and *CAT2* were significantly increased in MI than those in all other groups (except the EP0 group, *P* < 0.05).

**Conclusion:**

When FM was replaced by 30 g/kg of EPBM, growth performance, antioxidant capacity, and the ability to transport amino acids and peptides of hybrid grouper were significantly improved.

## Introduction

Fish meal (FM) is considered a high-quality feed material for aquatic animals due to its balanced amino acid composition, high protein content, rich in unsaturated fatty acids, and absence of anti-nutritional factors (1). The rapid expansion of the global aquaculture industry and the long-term transitional fishing of fisheries have led to a decrease in FM production year by year (2). The price of FM is rising, which severely restricted sustainable aquaculture development (3). In the past 10 years, replacing FM with alternative protein ingredients in aqua-feed has been recognized as a way (4) for the successful reduction in FM content in feed by using less expensive vegetable protein (5, 6). However, the absence of some essential amino acids (7), the high level of anti-nutritional factors (8), and the low digestibility (9) of plant protein resources have prevented their extensive use in feeds (10–12). Therefore, finding a cost-effective, safe, and healthy alternative source of protein has become one of the effective ways to solve the FM shortage problem.

Poultry by-product meal (PBM) is an important source of animal protein produced by the by-products produced (muscles of the tibia) during poultry processing through cooking, pressing, drying, and pulverizing. With the exception of methionine and lysine, PBM has a similar composition of amino acids to FM, has a high protein content, and is a valuable feed ingredient (13). Studies have reported that the use of moderate amounts of PBM replacement in aquafeeds achieved positive results on gilthead seabream (*Sparus aurata*) (14), striped bass (*Morone chrysops* × *M. saxatilis*) (15), cuneate drum (*Nibea miichthioides*) (16), cobia (*Rachycentron canadum*) (17), and turbot (*Scophthalmus maeoticus*) (18). However, a higher proportion of PBM replacing FM could reduce the growth performance (19, 20). The main problem with PBM replacing FM is the lack of restrictive amino acids and poor palatability (21). Compared with FM, PBM, lacking lysine and methionine, can affect its amino acid utilization and then inhibit fish growth (13). The replacement of FM with PBM can reduce the palatability of the feed, and it is the main reason for the significant reduction in the growth of fish (22, 23).

Further refinement of the PBM (such as enzyme digestion) can enhance nutrient availability and optimize the nutritional structure (24). The enzyme-digested poultry by-product meal (EPBM) was enriched with various peptides and free amino acids (25). The absorption of small peptides has the characteristics of fast transfer speed, low energy consumption, and difficulty to saturate the carrier (26). In addition, small peptides can be directly absorbed to avoid competition with amino acids, promote protein synthesis (27), accelerate the absorption of mineral elements (28), and improve the palatability of feeds (29), which can greatly improve economic benefits.

Grouper production, ranking third in marine cultured fish, reached 159,579 tons in 2021 (30). In China, the grouper (*Epinephelus fuscoguttatus* ♀ × *E. lanceolatus* ♂) is a marine fish of economic importance (31). The research into hybrid grouper has received a great deal of attention due to its advantages of growth performance, survival rate, and delicious taste (32). Therefore, this experiment investigated the effects of EPBM on growth, intestinal microbiota and morphology, non-specific immunity, and expression of amino acid and peptide transporters in hybrid grouper.

## Materials and methods

### Experimental diets

Seven trial diets (Table 1) were prepared with 0 g/kg (EP0), 30 g/kg (EP3), 60 g/kg (EP6), 90 g/kg (EP9), 120 g/kg (EP12), 150 g/kg (EP15), and 180 g/kg (EP18) of EPBM. The ingredients were crushed, weighed, and mixed thoroughly. The 3-mm diameter pellets were made using a twin-screw extruder (MY56 × 2A). The pellets were dehydrated at 25°C for approximately 36 h, at which point the moisture contents of the pellets were around 100 g/kg. The feed pellets were stored at −20°C.

**TABLE 1:** Formulation and proximate composition of the experimental diets (g/kg, dry matter).

Ingredient	EP0	EP3	EP6	EP9	EP12	EP15	EP18
Fish meal ^1^	360.0	330.0	300.0	270.0	240.0	210.0	180.0
EPBM ^2^	–	30.0	60.0	90.0	120.0	150.0	180.0
Soybean meal ^3^	275.0	180.0	180.0	180.0	180.0	180.0	180.0
Rapeseed meal ^4^	–	100.0	100.0	100.0	100.0	100.0	100.0
Wheat gluten flour ^6^	49.0	45.0	36.0	27.0	18.0	9.0	–
Spray-dried blood ^5^	30.0	30.0	30.0	30.0	30.0	30.0	30.0
Wheat meal ^7^	165.0	165.0	165.0	165.0	165.0	165.0	165.0
L-Lysine	7.1	7.6	7.9	8.2	8.4	8.7	8.9
DL-Methionine	3.5	3.5	3.7	3.9	4.1	4.4	4.6
Soybean oil	25.0	25.0	25.0	25.0	25.0	25.0	25.0
Fish oil	35.8	38.4	40.8	43.3	45.7	48.2	50.6
Soybean lecithin	15.0	15.0	15.0	15.0	15.0	15.0	15.0
Vitamin premix ^8^	2.0	2.0	2.0	2.0	2.0	2.0	2.0
Mineral premix ^8^	5.0	5.0	5.0	5.0	5.0	5.0	5.0
Vitamin C	0.5	0.5	0.5	0.5	0.5	0.5	0.5
Ca(H_2_PO_4_)_2_	15.0	15.0	15.0	15.0	15.0	15.0	15.0
Choline chloride	3.0	3.0	3.0	3.0	3.0	3.0	3.0
Ethoxyquin	0.3	0.3	0.3	0.3	0.3	0.3	0.3
Y_2_O_3_	1.0	1.0	1.0	1.0	1.0	1.0	1.0
Phytases	1.0	1.0	1.0	1.0	1.0	1.0	1.0
Microcrystalline cellulose	6.8	0.7	6.8	12.8	19.0	25.0	31.1
Total	1000.0	1000.0	1000.0	1000.0	1000.0	1000.0	1000.0
**Proximate composition ^9^**							
Moisture	102.7	105.3	102.5	107.4	100.4	102.9	102.2
Crude protein	498.2	494.1	496.5	500.4	501.3	499.1	500.3
Ether extract	104.8	103.5	105.9	105.1	106.9	107.4	109.0
Ash	93.9	92.3	90.3	87.3	84.2	81.2	78.2

^1^Fish meal: crude protein, 709.3 g/kg; ether extract, 80.6 g/kg.^2^EPBM, enzyme-digested poultry by-product meal: crude protein, 947.7 g/kg; ether extract, <0.2 g/kg.^3^Soybean meal: crude protein, 499.1 g/kg; ether extract, 14.8 g/kg.^4^Rapeseed meal: crude protein, 431 0 g/kg; ether extract, 12.5 g/kg.^5^Spray-dried blood: crude protein, 955.3 g/kg; ether extract, 6.5 g/kg.^6^Wheat gluten flour: crude protein, 794.3 g/kg; ether extract, 3.2 g/kg.^7^Wheat meal: crude protein, 128.4 g/kg; ether extract, 5.9 g/kg.^8^Vitamin premix and mineral premix were provided by Qingdao Master Biotech Co., Ltd, Qingdao, China.^9^Proximate compositions were measured values.

### Feeding trial

Hybrid groupers were purchased from the Zhanjiang grouper fry farm of Guangdong province, China. The groupers were domesticated for a fortnight to adapt to the experimental environment. During this period, industrial feeds with 500 g/kg crude protein were used to feed groupers. A total of 630 hybrid groupers (7.50 ± 0.02 g) were randomly divided into 7 groups, with three tanks (300 L) per group and 30 groupers in each tank. At 8:00 and 16:00 each day, groupers were fed until they were full for a cumulative period of 8 weeks. To ensure a consistent environment, each tank was replaced with approximately 210 L of water per day. During the experimental period, pH was 6.8–7.3, salinity was 28–31 g/L, the temperature range was 29.0–31.0°C, dissolved oxygen was not less than 5.0 mg/L, and ammonia nitrogen was not above 0.06 mg/L. All groupers and caretaking procedures were approved by the Institutional Animal Care and Use Committee of Guangdong Ocean University (Zhanjiang, China), in accordance with NIH guidelines (NIH Pub. No. 85 to 23, revised 1996).

### Sample collection and analysis

To obtain basal body metabolism, a 24-h fast was imposed on all groupers at the end of the 8-week culture experiment. MS-222 (1:10,000) was used to anesthetize groupers prior to sample collection. Groupers were weighed and counted to calculate survival rate (SR), feed coefficient rate (FCR), intaking feed rate (IFR), special growth rate (SGR), protein efficiency rate (PER), and weight gain rate (WGR). Three groupers were randomly selected, weighed, and measured their length to determine condition factor (CF), hepatopancreas and viscera were taken and weighed to determine the hepatopancreas somatic indices (HSI), and viscera somatic index (VSI). Three fish from each tank were used to determine the nutrient composition and to calculate the deposition rate of lipid and protein (LDR and PDR).

**FIGURE 1:**
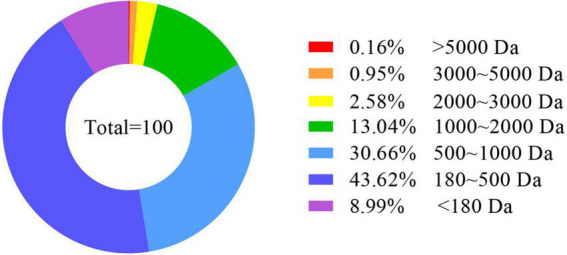
Peptide molecular weight distribution of enzyme-digested poultry by-product meal. Da, Dalton.

The AOAC (33) was used for analyzing whole-body and feed nutrient composition. The moisture content was determined by weight to a constant weight. Determination of ether extract (EE) by constant weight was performed after ether extraction. The Kjeldahl method was used in the analysis of crude protein (CP). The distribution of peptide molecular weight of EPBM (Figure 1) was analyzed using the national standard method (GB/T 22729-2008). During the feeding trial, after the groupers had been feeding for 4 h, feces were collected and used to calculate apparent digestibility coefficients for phosphorus (ADC _*P*_), calcium (ADC _*Ca*_), crude protein (ADC _*CP*_), and dry matter (ADC _*DM*_).

A 1-ml sterile syringe was used to collect blood samples from the spine of three groupers in each tank. After storage at 4°C for 12 h, the serum was collected by centrifugation (4,000 *g*) at 4°C for 15 min. The kit developed by Nanjing Jiancheng Bioengineering Institute (Nanjing, China) was used to measure total cholesterol (CHOL), total triglyceride (TG), glucose (GLU), albumin (Alb), total protein (TP), acid phosphatase (ACP), alkaline phosphatase (AKP), glutamic pyruvic transaminase (GPT), catalase (CAT), glutamic oxaloacetic transaminase (GOT), and superoxide dismutase (SOD) in serum.

Three fish from each tank were used to collect the distal intestine (DI), mid-intestine (MI), and proximal intestine (PI), and they were preserved in paraformaldehyde solution. The samples were dyed with hematoxylin and eosin, sectioned, and measured for muscle thickness (MT), villus width (VW), villus length (VL), crypt depth (CD), and the value of VL/CD.

The livers and intestines of three fish in each tank were collected. They were kept at −20°C for testing the enzyme activities. After wiping the outer skin of the intestines of three groupers in each tank with 75% alcohol, the intestines were placed at −80°C for the detection of microbial community. The PI, MI, and DI of three fish in each tank were stocked in RNA-later (Ambion, United States), and posteriorly placed at −80°C to analyze the expression of mRNA.

### Intestinal microbiota

The NucleoSpin^®^ Soil kits (Macherey-Nagel, Germany) were used to extract the bacterial DNA. The V3 + V4 of bacterial 16S rDNA genes were amplified by using universal primers. Amplification reactions were performed in a system containing 5 μL of KOD FX Neo Buffer, 2 μL of dNTP, 0.2 μL of KOD FX Neo, 0.3 μL of each primer, 50 ng bacterial DNA, and sterilized double-distilled water. The target region PCR was carried out at 95°C for 5 min; then 25 cycles of 30 s at 95°C, 30 s at 50°C, and 40 s at 72°C; and 72°C for 7 min. The Solexa PCR was performed in a system containing 10 μL of 2 × Q5 HF MM, 5 μL of target region PCR, and 2.5 μL of MPPI-a and MPPI-b, separately. The Solexa PCR was performed for 30 s at 98°C; then 10 cycles at 98°C for 10 s, 65°C for 30 s, and 72°C for 30 s; and lastly 5 min at 72°C. Following purification of the amplification products, sequencing and construction were performed on the sequencing platform. Sequencing results were classified into operational taxonomic units (OTUs). Based on relative abundance per OTU and the total number of OTUs, the alpha diversity index (such as abundance-based coverage estimator, ACE; Chao1; Shannon index; Simpson index; and Good’s coverage) and beta diversity index (such as analysis of similarities, ANOSIM; and principal coordinates analysis, PCoA) were calculated using Mothur (version 1.3.0) and QIIME (v. 1.7.0), respectively. Detections of differentially marked species were analyzed using line discriminant analysis effect size (LEfSe).

### Relative mRNA expression

The mRNA expression of oligopeptide transporter (*PepT1*), amino acid transporter of B^0,+^-type (*B^0, +^AT*, SLC6A19), sodium-coupled neutral amino acid transporter 3 (*SNAT3*, SLC38A3), excitatory amino acid transporter 1 (*EAAT1*, SLC1A3), sodium-dependent neutral amino acid transporter B^0^AT2-like (*B^0^AT2*, SLC6A15), cationic amino acid transporter 2 (*CAT2*, SLC7A2), proton-coupled amino acid transporter 1 (*PAT1*, SLC36A1), lysosomal amino acid transporter 1 (*LAAT1*, SLC38A9), and cationic amino acid transporter 1 (*CAT1*, SLC7A1) in the intestines were tested. These are shown in Table 2, which lists the sequences of exon boundary-spanning primers for the genes. The β-actin gene was used as a housekeeping gene, and the control group was used as the reference group. TRIzol reagent (Beijing Trans Gen Biotech Co., Ltd., China) was designed to produce total RNA from PI, MI, and DI. The DNA pollution in the RNA extract was removed by Recombinant DNase I (Takara, Japan). High purity of the RNA extract was indicated when the absorbance ratio at 260/280 nm on the spectrophotometer was 1.86 to 2.00. The Script™ reagent Kit (Takara, Japan) was used to reversely transcribed cDNA. The Premix Taq™ II Kit (Takara, Japan) was utilized for quantitative real-time PCR (qRT-PCR). The qRT-PCR was performed in a 10-μL volume with 3.2 μL of sterilized double-distilled water, 0.4 μL of each primer, 1 μL of cDNA, and 5 μL of Master Mix (Bio-Rad Labs). The qRT-PCR was performed under the procedure of Yang et al. (34). According to the method of Mu et al. (35) for 2^–Δ Δ^ *^CT^*, expressions of genes were analyzed.

**TABLE 2:** Sequence of the primers used for quantitative real-time PCR.

Target gene	Primer sequence (5′-3′)	Annealing temperature (°C)	Length of amplified product (bp)
β*-actin*	F: TGCGTGACATCAAGGAGAAGC	61	151
	R: TCTGGGCAACGGAACCTCT	60	
*CAT1*	F: GCTACCTGCTTCTATGCCTTTG	59	116
	R: AAGCAGATGAGCAGCGAGGA	60	
*CAT2*	F: CCTACTCGCTGGTTGCTGTG	59	87
	R: CTCTCTCACTGGTGCCTGCTC	60	
*LAAT1*	F: TTAGGATGGACGACTGAAGGAA	59	95
	R: CTCACATCTTGGGCACATTCC	60	
*PAT1*	F: GGGAAAACAGACGGTGAACTT	58	141
	R: GTGTTGTTGATGTGGCAGTTGA	59	
*B^0^AT2*	F: GCTCAACTTAGGACTCAGCACCAT	61	76
	R: AGTTTTGAAGCGGTCGGTGA	60	
*SNAT3*	F: ACTGAACTGAGCAACCCAACC	59	114
	R: GTAGAAGGTCAGATAGCCAAACG	58	
*EAAT1*	F: GGTTTCTGGACCGATTGCG	61	138
	R: TCTTGTCTGCCTCCTCCACC	60	
*B^0, +^AT*	F: TGTCTACATCCCCTTCATCCATTAC	61	91
	R: AGGGGCGACCTCTAAGAACAG	60	
*PepT1*	F: TGCTGCTGTTCGGGTGCT	60	79
	R: CGGGCTTCTTCCTGGGTT	60	

*CAT1*: cationic amino acid transporter 1; *CAT2*: cationic amino acid transporter 2; *LAAT1*: lysosomal amino acid transporter 1; *PAT1*: proton-coupled amino acid transporter 1; *B*^0^*AT2*: sodium-dependent neutral amino acid transporter B^0^AT2-like; *SNAT3*: sodium-coupled neutral amino acid transporter 3; *EAAT1*: excitatory amino acid transporter 1; *B*^0,+^*AT*: B^0,+^-type amino acid transporter; *PepT1*: oligopeptide transporter.

### Statistics and calculations

The following formula was used to calculate growth performance:

Intaking feed rate (IFR,%/d) = W_*d*_/(t × (W_*f*_ + W_*i*_)/2) × 100

Protein efficiency rate (PER) = (W_*f*_ - W_*i*_)/W_*f*_ × CP_*d*_

Special growth rate (SGR,%/d) = [ln(W_*f*_) -ln(W_*i*_)]/t × 100

Weight gain rate (WGR,%) = (W_*f*_ - W_*i*_)/W_*i*_ × 100

Feed coefficient rate (FCR) = W_*d*_/(W_*f*_ - W_*i*_)

Survival rate (SR,%) = N_*f*_/N_*i*_ × 100

Condition factor (CF,%) = W_*b*_/(L_*b*_)^3^ × 100

Hepatopancreas somatic indices (HSI,%) = 100 × W_*h*_/W_*b*_

Viscera somatic index (VSI,%) = 100 × W_*v*_/W_*b*_

Protein deposition rate (PDR,%) = (W_*f*_ × CP_*f*_ – W_*i*_ × CP_*i*_)/(W_*d*_ × CP_*d*_) × 100

Lipid deposition rate (LDR,%) = (W_*f*_ × EE_*f*_ – W_*i*_ × EE_*i*_)/W_*d*_ × EE_*d*_ × 100

Apparent digestibility coefficient of dry matter (ADC_*DM*_,%) = (1 – (Y_*d*_/Y_*f*_)) × 100

Apparent digestibility coefficient of nutrients (ADC_*N*_,%) = (1 – (N_*ff*_/N_*d*_) × (Y_*d*_/Y_*f*_)) × 100

where t is the days of the feeding experiment, W_*i*_ (g) is the mean initial body weight, W_*f*_ (g) is the mean final body weight, W_*d*_ (g) is the diet weight of each groups fish intaking (dry weight), CP_*d*_ (%) is the percentage of crude protein of diet, N_*i*_ is the fish amount of initial test, N_*f*_ is the fish amount of final test, L_*b*_ (cm) is the body length of the single fish, W_*b*_ (g) is the body weight of the single fish, W_*v*_ (g) is the viscera weight of the single fish, W_*h*_ (g) is the hepatopancreas weight of the single fish, CP_*f*_ (%) is the percentage of crude protein of fish body of final test, CP_*i*_ (%) is the percentage of crude protein of fish body of initial test, EE_*f*_ (%) is the percentage of ether extract of fish body of final test, EE_*i*_ (%) is the percentage of ether extract of fish body of initial test, EE_*d*_ (%) is the percentage of ether extract of diet, Y_*d*_ (%) is the percentage of Y_2_O_3_ of diet, Y_*f*_ (%) is the percentage of Y_2_O_3_ of feces, N_*ff*_ (%) is the percentage of nutrients of feces, and N_*d*_ (%) is the percentage of nutrients of diet.

An ANOVA (one-way analysis of variance) was used for the statistical analysis of all original data. Comparison of means among treatments using Tukey’s test. SPSS 22.0 was used to conduct all statistical analyses. The expression of all data as means ± SEM. When *P*-values were <0.05, the difference was considered to be significant. Different letters indicate significant differences among groups (a > b > c > d).

## Results

### Growth performance

The FBW, WGR, SGR, and HSI in the EP3 group were significantly better than those in the EP0, EP12, EP15, and EP18 groups (*P* < 0.05). With the increase in EPBM content, PER was gradually decreased and was the lowest in the EP18 group (*P* < 0.05). The trend of FCR and IFR was opposite to that of PER. The FCR and IFR were significantly higher in the EP18 group than that in the EP0, EP3, and EP6 groups (*P* < 0.05). The LDR showed a maximum value when the feed was supplemented with EPBM at a level of 60 g/kg, which was significantly higher than the group with EPBM additions of 90–180 g/kg (*P* < 0.05). The SR, CF, VSI, and PDR were not significantly influenced by diet (*P* > 0.05; Table 3).

**TABLE 3:** Growth and apparent digestibility coefficients of hybrid groupers fed with diets.

Index	EP0	EP3	EP6	EP9	EP12	EP15	EP18	SEM	*P*-values
IBW (g)	7.51	7.50	7.50	7.49	7.49	7.51	7.49	0.01	0.763
FBW (g)	65.50*^b^*	73.27*^a^*	68.87*^ab^*	68.23*^b^*	67.87*^b^*	66.30*^b^*	60.37*^c^*	0.87	<0.001
WGR (%)	771.94*^b^*	876.90*^a^*	817.71*^ab^*	810.73*^b^*	805.67*^b^*	782.92*^b^*	705.91*^c^*	11.57	<0.001
SGR (%/d)	3.87*^b^*	4.07*^a^*	3.96*^ab^*	3.95*^ab^*	3.93*^b^*	3.89*^b^*	3.73*^c^*	0.02	<0.001
PER	2.55*^a^*	2.64*^a^*	2.62*^a^*	2.52*^a^*	2.41*^ab^*	2.38*^ab^*	2.22*^b^*	0.04	0.001
IFR (%/d)	2.23*^b^*	2.23*^b^*	2.21*^b^*	2.27*^ab^*	2.38*^ab^*	2.40*^ab^*	2.51*^a^*	0.03	0.013
FCR	0.79*^b^*	0.77*^b^*	0.77*^b^*	0.79*^b^*	0.83*^ab^*	0.84*^ab^*	0.90*^a^*	0.01	0.003
SR (%)	98.89	95.56	94.44	100.00	94.45	94.45	98.89	0.69	0.169
CF (%)	2.80	2.72	2.71	2.61	2.63	2.65	2.62	0.03	0.580
VSI (%)	9.95	10.80	10.68	10.47	10.19	10.28	10.11	0.11	0.387
HSI (%)	2.75*^b^*	3.29*^a^*	2.37*^b^*	2.48*^b^*	2.44*^b^*	2.29*^b^*	2.38*^b^*	0.08	<0.001
PDR (%)	28.89	32.05	33.38	30.62	31.40	29.05	28.67	0.51	0.057
LDR (%)	46.65*^abc^*	54.48*^ab^*	56.96*^a^*	43.94*^bc^*	43.43*^bc^*	42.61*^c^*	37.61*^c^*	1.62	0.001
ADC _*DM*_	78.70*^ab^*	78.77*^ab^*	79.82*^a^*	74.77*^c^*	77.12*^b^*	74.66*^c^*	73.40*^c^*	0.53	<0.001
ADC _*CP*_	93.10*^a^*	92.69*^ab^*	92.86*^ab^*	91.05*^cd^*	92.24*^b^*	91.18*^c^*	90.43*^d^*	0.22	<0.001
ADC _*Ca*_	17.23*^c^*	29.32*^a^*	23.82*^b^*	10.31*^d^*	31.15*^a^*	11.01*^d^*	7.26*^d^*	2.01	<0.001
ADC _*P*_	49.58*^b^*	55.21*^ab^*	54.47*^ab^*	47.33*^b^*	57.71*^a^*	48.20*^b^*	50.849*^ab^*	0.97	0.004

Mean values ± SEM are presented for each group (n = 3); different superscript letters indicate significant differences among groups (a > b > c > d; P < 0.05).IBW, initial body weight; FBW, final body weight; WGR, weight gain rate; SGR, specific growth rate; PER, protein efficiency rate; IFR, intaking feed rate; FCR, feed conversion rate; SR, survival rate; CF, condition factor; VSI, viscera somatic index; HSI, hepatopancreas somatic indices; PDR, protein deposition rate; LDR, lipid deposition rate. ADC *_DM_*, apparent digestibility coefficient for dry matter of the dietary; ADC *_CP_*, apparent digestibility coefficient for crude protein of the dietary; ADC *_Ca_*, apparent digestibility coefficient for calcium of the dietary; ADC *_P_*, apparent digestibility coefficient for phosphorus of the dietary.

### Apparent digestibility coefficient

The ADC _*DM*_ in the EP6 group, the ADC _*CP*_ in the EP0 group, and the ADC _*Ca*_ in the EP3 group were considerably larger than those in the EP9, EP15, and EP18 groups (*P* < 0.05; Table 3). Compared with the EP0 group, the ADC _*P*_ was substantially higher in the EP12 group (*P* < 0.05).

### Whole-body composition

In the EP12 group, CP content was significantly best, except in the EP18 group (*P* < 0.05). The EE content in the EP6 group was considerably higher than that in the EP9 and EP18 groups (*P* < 0.05). Lower ash content in the group uses EPBM instead of FM compared to the EP0 group (*P* < 0.05). The moisture was not significantly impacted by the experimental diet (*P* > 0.05; Table 4).

**TABLE 4:** Whole-body composition, serum biochemical indices, and enzyme activities of hybrid groupers fed with diets.

Index	EP0	EP3	EP6	EP9	EP12	EP15	EP18	SEM	*P*-values
**Whole body composition**									
Moisture (g/kg)	825.3	821.7	819.7	825.5	821.7	829.0	823.1	1.1	0.148
CP (g/kg)	614.2*^d^*	611.9*^d^*	629.6*^bc^*	619.4*^cd^*	650.8*^a^*	632.3*^bc^*	638.3*^ab^*	3.0	<0.001
EE(g/kg)	221.2*^ab^*	238.2*^ab^*	250.3*^a^*	208.9*^b^*	223.4*^ab^*	222.0*^ab^*	207.1*^b^*	4.2	0.030
Ash (g/kg)	148.9*^a^*	120.9*^cd^*	114.5*^d^*	137.1*^b^*	113.1*^d^*	126.7*^bc^*	120.0*^cd^*	2.8	<0.001
**Biochemical indices**									
TP (g/L)	57.37*^ab^*	56.33*^ab^*	56.77*^ab^*	56.00*^ab^*	62.77*^a^*	46.67*^b^*	49.02*^b^*	1.33	0.003
Alb (g/L)	8.51*^b^*	11.99*^a^*	11.34*^ab^*	12.56*^a^*	14.43*^a^*	12.69*^a^*	11.97*^a^*	0.43	0.001
CHOL (mmol/L)	2.73	2.96	2.74	3.21	2.56	2.91	2.69	0.09	0.589
TG (mmol/L)	0.52*^d^*	0.50*^d^*	0.55*^cd^*	0.57*^bc^*	0.63*^abc^*	0.66*^ab^*	0.70*^a^*	0.17	<0.001
GLU (mmol/L)	3.77*^bc^*	2.91*^c^*	4.21*^ab^*	3.93*^ab^*	4.76*^a^*	3.40*^bc^*	3.98*^ab^*	0.14	<0.001
**Enzyme activities**									
SOD (U/mL)	17.50*^b^*	23.53*^a^*	19.09*^b^*	19.54*^b^*	16.49*^b^*	17.40*^b^*	17.49*^b^*	0.55	0.001
CAT (U/mL)	2.71*^ab^*	2.79*^a^*	2.62*^ab^*	2.60*^ab^*	2.67*^ab^*	2.24*^b^*	2.50*^ab^*	0.05	0.049
GOT (U/L)	22.50*^ab^*	29.88*^a^*	23.60*^ab^*	25.39*^ab^*	25.59*^ab^*	18.92*^b^*	21.88*^ab^*	0.90	0.015
GPT (U/L)	20.00*^a^*	17.39*^ab^*	14.54*^bcd^*	14.27*^bcd^*	12.21*^cd^*	11.71*^d^*	15.47*^bc^*	0.65	<0.001
AKP (U/L)	16.94*^a^*	14.33*^bc^*	16.69*^ab^*	14.87*^abc^*	14.76*^abc^*	14.83*^abc^*	14.05*^c^*	0.28	0.005
ACP (U/L)	10.61*^ab^*	11.60*^a^*	11.28*^ab^*	12.54*^a^*	12.06*^a^*	10.03*^ab^*	8.76*^b^*	0.32	0.004

Mean values ± SEM are presented for each group (n = 3); different superscript letters indicate significant differences among groups (a > b > c > d; P < 0.05). Moisture is the value on wet matter, and other whole-body compositions are values on dry matter.CP, crude protein; EE, ether extract. TP, total protein; Alb, albumin; CHOL, total cholesterol; TG, total triglyceride; GLU, glucose, SOD, superoxide dismutase; CAT, catalase; GOT, glutamic oxaloacetic transaminase; GPT, glutamic pyruvic transaminase; AKP, alkaline phosphatase; ACP, acid phosphatase.

### Serum biochemical indices

There was no considerable variation in CHOL (*P* > 0.05; Table 4). The EPBM substitution of FM resulted in significantly higher serum Alb levels in grouper (*P* < 0.05, except in the EP6 group). As the proportion of FM was replaced by EPBM increased, the TG content gradually increased, with a maximum value in the EP18 group (*P* < 0.05). The TP and GLU content in the EP12 group was substantially greater than that in the EP15 group (*P* < 0.05).

### Enzyme activity index in serum

The CAT, GOT, and SOD activities in the EP3 group were prominently greater than in the EP15 group (*P* < 0.05; Table 4). As the proportion of FM replaced by EPBM was increased, GPT and AKP activity was significantly decreased (*P* < 0.05). ACP activities were markedly greater in the EP3, EP9, and EP12 groups than those in the EP18 group (*P* < 0.05).

### Digestive enzyme activities

In the intestine, the chymotrypsin, lipase, and trypsin activities were prominently increased in the EP12 and EP15 groups compared to the EP0 group (*P* < 0.05). Amylase activities in the EP12 and EP18 groups were prominently increased than those in the EP6 group (*P* < 0.05). In the liver, compared with the EP0 group, the chymotrypsin and amylase activities were substantially increased by adding 120 g/kg of EPBM (*P* < 0.05), with significantly greater activity of trypsin in the EP3 group than that in the EP0 group (*P* < 0.05). The lipase was not significantly different (*P* > 0.05; Table 5).

**TABLE 5:** Intestinal and liver digestive enzyme activities of hybrid groupers fed with diets.

Index	EP0	EP3	EP6	EP9	EP12	EP15	EP18	SEM	*P*-values
**Intestinal**									
Chymotrypsin (IU/g)	1.66*^d^*	2.42*^c^*	2.55*^bc^*	3.11*^ab^*	3.33*^a^*	3.30*^a^*	2.68*^bc^*	0.13	<0.001
Lipase (U/g)	3.56*^c^*	3.74*^c^*	4.67*^b^*	5.73*^a^*	5.82*^a^*	5.71*^a^*	6.09*^a^*	0.23	<0.001
Amylase (IU/g)	2.55*^ab^*	2.71*^ab^*	2.30*^b^*	2.74*^ab^*	3.16*^a^*	2.55*^ab^*	3.14*^a^*	0.08	0.018
Trypsin (U/mg)	13.58*^b^*	12.41*^b^*	13.38*^b^*	17.64*^a^*	17.64*^a^*	17.25*^a^*	14.05*^b^*	0.49	<0.001
**Liver**									
Chymotrypsin (IU/g)	1.75*^b^*	2.44*^a^*	2.26*^ab^*	1.78*^b^*	2.40*^a^*	2.02*^ab^*	2.57*^a^*	0.08	0.001
Lipase (U/g)	4.62	5.39	4.11	4.18	5.08	5.13	5.23	0.14	0.031
Amylase (IU/g)	2.07*^b^*	2.06*^b^*	2.02*^b^*	2.15*^b^*	3.04*^a^*	2.26*^b^*	2.05*^b^*	0.09	0.005
Trypsin (U/mg)	8.57*^d^*	14.22*^a^*	13.30*^ab^*	12.32*^abc^*	11.33*^bcd^*	10.89*^bcd^*	9.57*^cd^*	0.45	<0.001

Mean values ± SEM are presented for each group (n = 3); different superscript letters indicate significant differences among groups (a > b > c > d; P < 0.05).

### Intestinal morphology

**FIGURE 2:**
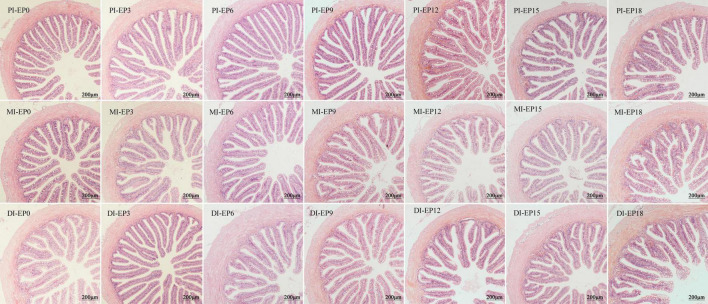
Light microscopy (20×) of the proximal intestine (PI), mid-intestine (MI), and distal intestine (DI) morphology of hybrid groupers fed with diets. Scale bar: 200 μm.

There was no obvious difference in the MT of PI, VW, MT, and VL/CD value of MI, as well as VL and VW of DI (*P* > 0.05; Figure 2 and Table 6). The MT and CD of DI, as well as VW and VL of PI in the EP15 group, were substantially greater than that in the EP0 and EP3 groups (*P* < 0.05). The VL and CD of MI were significantly higher in the EP18 group (*P* < 0.05). The CD of DI in the EP18 group was significantly higher than that in all other groups, except for the EP15 group (*P* < 0.05). The VL/CD values of PI and DI in the EP3 group were significantly greater than those in the EP15 group (*P* < 0.05).

**TABLE 6:** Intestinal morphology of hybrid groupers fed with diets.

Index(μ m)	EP0	EP3	EP6	EP9	EP12	EP15	EP18	SEM	*P*-values
**PI**									
VL	448.60*^c^*	512.83*^bc^*	558.82*^abc^*	572.23*^abc^*	599.92*^ab^*	667.29*^a^*	530.68*^abc^*	17.36	0.007
VW	70.36*^b^*	66.44*^b^*	74.08*^b^*	72.59*^b^*	77.72*^ab^*	99.16*^a^*	82.17*^ab^*	2.66	0.004
MT	82.28	104.71	94.16	88.95	103.24	92.73	90.84	2.63	0.259
CD	38.07*^c^*	35.16*^c^*	45.29*^bc^*	45.01*^bc^*	49.72*^ab^*	58.78*^a^*	40.46*^bc^*	1.79	<0.001
VL/CD	11.80*^ab^*	14.60*^a^*	12.32*^ab^*	12.73*^ab^*	12.08*^ab^*	11.49*^b^*	13.12*^ab^*	0.30	0.049
**MI**									
VL	433.61*^b^*	469.88*^b^*	470.69*^b^*	502.32*^b^*	451.85*^b^*	453.64*^b^*	633.97*^a^*	15.94	0.001
VW	71.82	76.99	82.10	76.76	87.93	80.50	87.91	1.81	0.123
MT	105.49	98.86	104.31	105.41	103.73	99.18	112.37	1.41	0.158
CD	42.10*^b^*	48.73*^b^*	43.62*^b^*	45.66*^b^*	40.75*^b^*	48.69*^b^*	79.44*^a^*	2.92	<0.001
VL/CD	10.33	9.63	10.91	11.06	11.08	9.51	7.98	0.32	0.070
**DI**									
VL	545.55	537.49	574.13	512.63	541.77	536.34	555.84	8.86	0.752
VW	91.91	89.66	86.56	84.01	87.80	86.46	90.67	0.97	0.343
MT	103.31*^bc^*	95.98*^c^*	125.82*^abc^*	119.64*^abc^*	119.36*^abc^*	138.69*^a^*	128.46*^ab^*	3.67	0.005
CD	47.68*^c^*	41.47*^c^*	48.53*^c^*	41.31*^c^*	51.52*^bc^*	61.55*^ab^*	63.74*^a^*	1.98	0.006
VL/CD	11.44*^ab^*	12.96*^a^*	11.89*^ab^*	12.44*^ab^*	10.54*^bc^*	8.75*^c^*	8.74*^c^*	0.38	0.004

Mean values ± SEM are presented for each group (n = 3); different superscript letters indicate significant differences among groups (a > b > c; P < 0.05). PI, proximal intestine; MI, mid-intestine; DI, distal intestine; VL, villus length; VW, villus width; MT, muscle thickness; CD, crypt depth. The value of VL/CD has no unit.

### Intestinal microbiota

**FIGURE 3:**
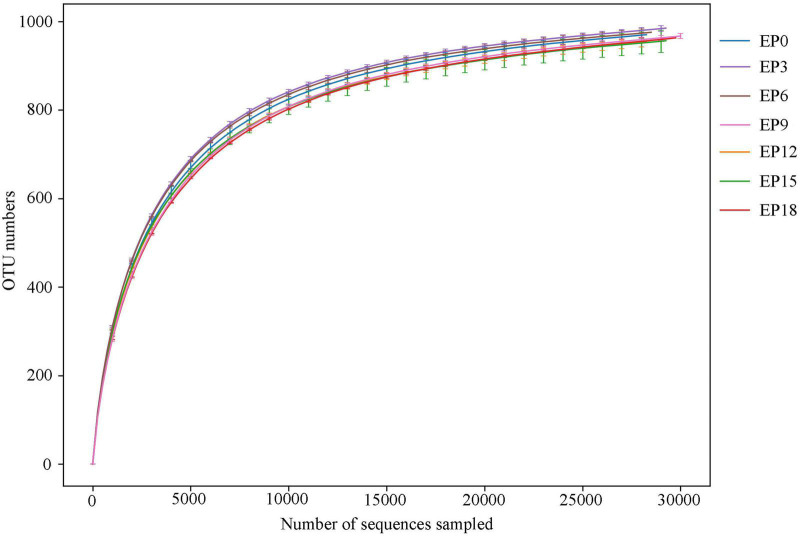
Rarefaction curve of the intestinal microbiota in hybrid groupers fed with diets.

The samples were sequenced, and 1,680,219 pairs of reads were acquired. Following filtration and splicing of double-end reads, 1,593,640 clean tags were produced. At least 74,041 clean tags were produced per sample, with an average of 75,888 clean tags. In all samples, the rarefaction curves of the OTU numbers were observed to have the same trend and gradually converged to a saturation plateau (Figure 3), indicating that the sequencing has been accomplished.

**FIGURE 4:**
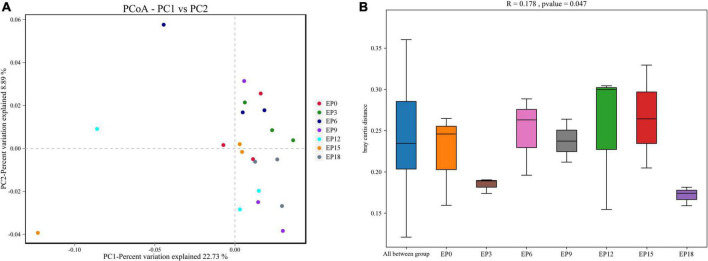
Principal coordinates analysis, PCoA **(A)**; and analysis of similarities, ANOSIM **(B)** of the intestinal microbiota in hybrid groupers fed with diets.

The coverage was not less than 0.9950, indicating that the sequencing depth has met the experimental requirements (Table 7). The Chao 1, ACE, Simpson index, Shannon index, Good’s coverage, and OTUs were not significantly impacted (*P* > 0.05; Table 7). The scattered sample of the seven experimental groups was basically concentrated on the right side of the coordinate axis (Figure 4A). ANOSIM showed that the *R*-value was 0.178, indicating that there was no major change among or within the groups. The *P*-value was 0.047, which was less than 0.05, indicating that the test result was highly reliable (Figure 4B).

**TABLE 7:** Numbers of OTUs and alpha diversity index of the intestinal microbiota in hybrid groupers fed with diets.

Index	EP0	EP3	EP6	EP9	EP12	EP15	EP18	SEM	*P*-values
OTUs	976.33	987.67	981.33	970.33	961.00	981.33	966.67	2.71	0.075
Good’s coverage	0.9957	0.9976	0.9978	0.9976	0.9975	0.9976	0.9974	0.0003	0.447
Shannon	5.13	5.11	5.20	5.01	5.30	5.26	5.01	0.18	0.208
Simpson	0.024	0.027	0.021	0.028	0.018	0.020	0.027	0.004	0.089
ACE	1001.06	989.01	988.28	996.59	1011.77	1004.37	996.60	9.96	0.163
Chao 1	1001.91	998.40	993.38	1007.74	1022.25	1009.30	1005.63	10.46	0.388

Mean values ± SEM are presented for each group (n = 3); no superscript letters indicate no significant difference among groups (P > 0.05).OTU, operational taxonomic unit; ACE, abundance-based coverage estimator.

**FIGURE 5:**
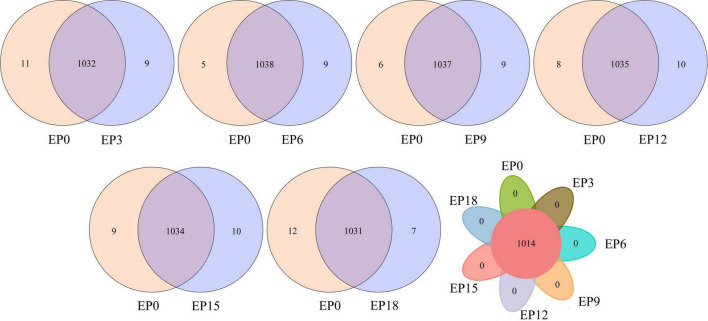
Venn diagram of unique and shared OTUs.

The Venn diagram shows the unique and shared OTUs (Figure 5). The number of the shared OTUs in the EP3, EP6, EP9, EP12, EP15, and EP18 groups was 1,032, 1,038, 1,037, 1,035, 1,034, and 1,031, and the number of unique OTUs was 9, 9, 9, 10, 10, and 7, respectively, compared to the EP0 group. Interestingly, in the experimental samples of seven groups, the shared OTU was 1,014, and there was no unique OTU.

**FIGURE 6:**
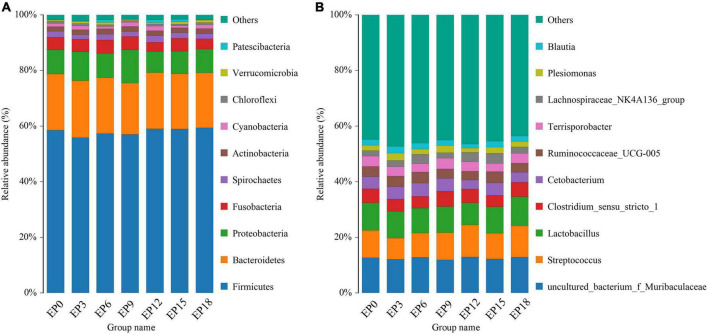
Taxonomy classification of reads at the phylum **(A)** and genus **(B)** taxonomic levels of the intestinal microbiota in hybrid groupers fed with diets.

The top 10 dominant bacteria in the intestine of hybrid grouper, at the phylum level, were Patescibacteria, Verrucomicrobia, Chloroflexi, Cyanobacteria, Actinobacteria, Spirochetes, Fusobacteria, Proteobacteria, Bacteroidetes, and Firmicutes, respectively (Figure 6A). The top 10 dominant bacteria at the genus level were *Lachnospiraceae_NK4A136_group, Blautia, Plesiomonas, Terrisporobacter, Ruminococcaceae_UCG-005, Clostridium Clostridium_sensu_stricto_1, Lactobacillus, Streptococcus*, and *uncultured_bacterium_f_Muribaculaceae*, respectively (Figure 6B).

**FIGURE 7:**
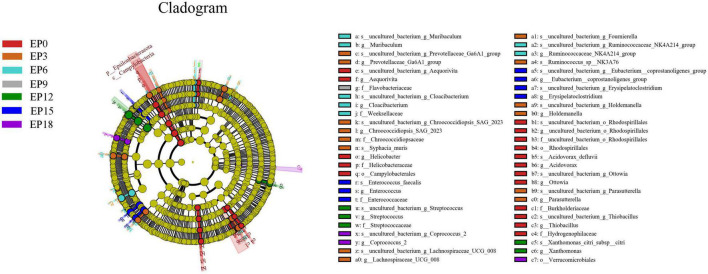
LEfSe analysis of the intestinal bacteria microbiota in hybrid groupers fed with diets. The circles radiating from the inside to the outside of the evolutionary branch diagram represent the classification levels of phylum (p), class (c), order (o), family (f), genus (g), and species (s).

The biomarkers with statistical differences in the EP0 group were *Helicobacter*, followed by *Acidovorax, uncultured_bacterium_o_Rhodospirillales, Aequorivita, Thiobacillus*, and *Ottowia*, according to LEfSe analysis (Figure 7). The biomarkers with statistical differences in the EP3 group were *Prevotellaceae_Ga6A1_group, Parasutterella, Helicobacter, Holdemanella, Lachnospiraceae_UCG_008, Chroococcidiopsis_SAG_2023, Ruminococcus*, and *Fournierella*. The biomarkers with statistical differences in the EP6 group were *Ruminococcaceae_NK4A214_group, Muribaculum*, and *Cloacibacterium*. The biomarker with statistical differences in the EP9 group was *Flavobacteriaceae*. The biomarkers with statistical differences in the EP12 group were *Streptococcus, Xanthomonas*, and *Enterococcus*. The biomarkers with statistical differences in the EP15 group were *Erysipelatoclostridium* and *Eubacterium__coprostanoligenes_group*. The biomarker with statistical differences in the EP18 group was *Coprococcus_2*.

### Relative mRNA expression

**FIGURE 8:**
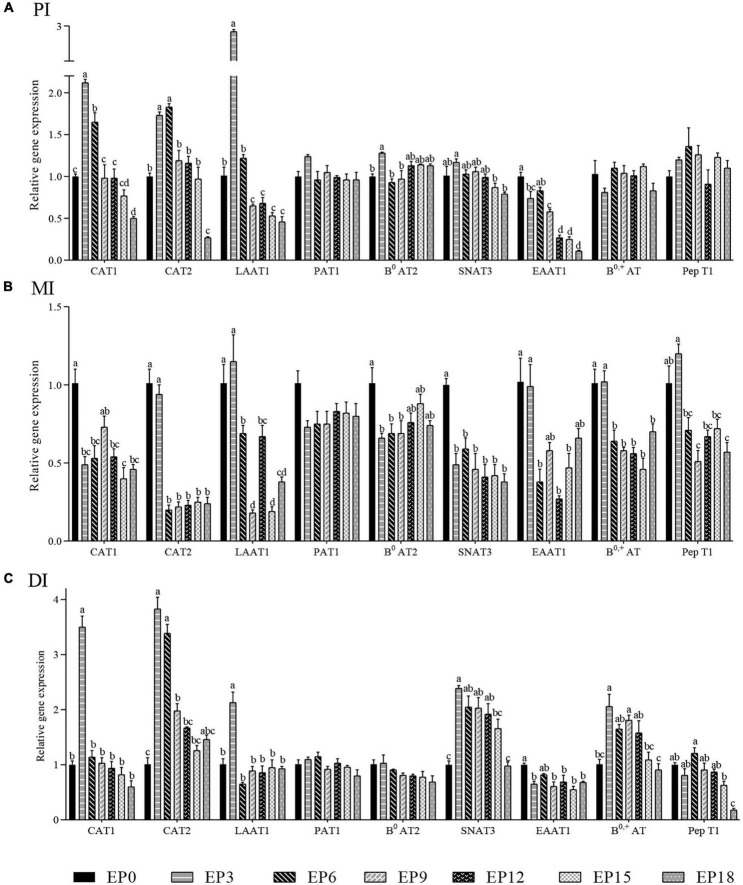
Relative mRNA expression of amino acid and small peptide-related transport in the **(A)** proximal intestine (PI), **(B)** mid-intestine (MI), and **(C)** distal intestine (DI) of hybrid groupers fed with diets, respectively. The β-actin gene was used as a housekeeping gene, and the control group was used as the reference group. Columns represented the mean values ± S. E. M. of each group. Different letters in each figure indicate significant differences among groups (a > b > c > d; *P* < 0.05).

In the PI, as the EPBM amount increased, the expressions of *SNAT3, B^0^AT2, LAAT1, CAT1, and CAT2* showed an increase followed by a decrease, and the expressions of the above five genes were significantly higher when the EPBM amount was 30 g/kg (*P* < 0.05). The *EAAT1* expression was prominently greater in the EP0 group, except for the EP6 group (*P* < 0.05). The experimental diet had no significant effect on the expressions of *PAT1, B^0, +^AT*, and *PepT1* (*P* > 0.05; Figure 8A).

In the MI, the expressions of *CAT2, LAAT1, EAAT1, B^0, +^AT*, and *PepT1* were not significantly dissimilar in the EP3 group from that in the EP0 group (*P* > 0.05). EPBM substitution of FM led to a significant reduction in the expression of *CAT1, B^0^AT2, and SNAT3* (*P* < 0.05). The effect of EPBM on the expression of *PAT1* was not significant (*P* > 0.05; Figure 8B).

In the DI, the expression of *B^0, +^AT, SNAT3, LAAT1, CAT2, and CAT1* gradually increased with increasing EPBM levels, showing a maximum in the EP3 group, followed by a gradual decrease (*P* < 0.05). The expression of *PepT1* in the EP6 group was prominently increased than that in the EP15 and EP18 groups (*P* < 0.05). There were no major changes in the expressions of *PAT1* and *B^0^AT2* (*P* > 0.05; Figure 8C).

## Discussion

In this research, the theoretical basis for the application of EPBM instead of FM in grouper feed was provided. The results showed that as the proportion of EPBM replacing FM increased, the LDR, HSI, SGR, and WGR of hybrid grouper tended to increase and then decrease, PER showed a downward trend, and IFR showed an upward trend. FM (from 360 to 210 g/kg) could be potentially replaced by 150 g/kg of EPBM in equal amounts without reducing growth performance. Studies indicated that PBM was replaced by more FM in the feed, the growth performance of giant croaker (*Nibea japonica*) (FM (from 400 to 240 g/kg) was replaced by 139 g/kg PBM) (35), hybrid striped bass (FM (from 250 to 162 g/kg) was replaced by 172 g/kg PBM) (19), and turbot (FM (from 773 to 380 g/kg) was replaced by 432 g/kg PBM) (36), which were significantly reduced. This may be caused by less FM content resulting in a weakening of the promotion effects of the attractants and growth-promoting factors (37).

In this experiment, replacing FM (from 360 to 330 g/kg) with an equivalent amount of 30 g/kg of EPBM significantly improved the growth performance, while the FM content was reduced to 210 g/kg, and growth performance was not reduced. This may be because 832.7 g/kg of the small peptides in the EPBM has a peptide molecular weight of less than 1,000 Da. In animals, small peptide proteins (dipeptide and tripeptide) can be directly absorbed (38). The uptake of peptides takes place through a special transport system, which has a fast absorption speed and low energy consumption, the carriers of peptide transport are not easy to saturate, and peptide transports are not competitive and inhibitory (39). This increases the rate of protein absorption, reduces the energy consumption of the animal to break down proteins, and saves energy for growth (40). Small peptides in feed have a significant role in promoting the growth of aquatic animals (41, 42) as well as improving the PDR and LDR of the hybrid grouper.

Amino acid balance is an important factor for growth (43). The supplementation of essential amino acids in this test was also one of the reasons why growth (the FM content was reduced to 210 g/kg) was not inhibited. The amino acid composition of EPBM was similar to FM. The supplementation of exogenous amino acids optimizes the amino acid structure and satisfies the nutritional requirements of the grouper. When FM was replaced by 180 g/kg of EPBM, WGR and SGR were reduced. It is possible that the excessive level of free amino acid is the cause. Excess amino acid produced by low molecular weight peptides in EPBM can cause competition of the transport mechanism and saturation of the transport system (44), restrict amino acid absorption, which is the main reason for the reduced growth, PER and ADC_CP_, and increase FCR in hybrid grouper (45). This suggests that more enzymatic protein contents in the feed may be detrimental to growth. Similar results were found in carp (46) and gilthead seabream (47).

Enzyme-digested poultry by-product meal is rich in flavor and taste-stimulating peptides (48). This may account for the gradual increase in IFR with the increase in EPBM content. With the increase in the proportion of FM replaced by EPBM, the HSI increased first and then decreased and showed a maximum value in the EP3 group. This may be because the WGR of the EP3 group was higher, and more protein and lipid were deposited in the liver so that the weight of the liver was greatly increased and finally leads to an increase in HSI.

The apparent digestibility of growing pigs was positively correlated with growth (49). In this experiment, the change of ADC_CP_ and ADC_DM_ was similar to the growth performance, and a significant decrease was found when FM was replaced by 90 g/kg of EPBM. ADC_CP_ of feed was between 70 and 89%, while FM was replaced by PBM in fish feed (50–52). The ADC_*CP*_ of the feed in this experiment was between 90.43 and 93.10%. It is closely related to the small peptides in EPBM which are easily digested and absorbed. Plus, the ADC _*CP*_ in the EP3 group was not substantially diverse from that in the EP0 group, which was also a factor for the better growth performance of the EP3 and EP6 groups.

Many studies have reported that the replacement of FM by PBM has no significant effect on the whole-body composition of yellow catfish (*Pelteobagrus fulvidraco*) (20), turbot (21), spotted rose snapper (*Lutjanus guttatus*) (37), Atlantic salmon (*Salmo salar*) (50), and crayfish (*Pacifastacus leniusculus* Dana, Astacidae) (53). However, replacing FM with a protein source containing small peptides has a significant effect on the whole-body composition of hybrid grouper (hydrolyzed krill meal) (54), turbot (peptides hydrolyzed from poultry by-products) (55), and yellow catfish (fish protein hydrolyzate) (56). This may be caused by the rich small peptides in the feed that change the absorption of protein and lipid. In this study, the moisture content of the whole body was not positively influenced. As the EPBM levels increase, CP and EE contents tended to increase first and then decrease. It was consistent with the trend of PDR and LDR. When FM was replaced by 60 g/kg of EPBM, LDR of hybrid grouper was the highest, and more lipid was deposited. This also resulted in higher EE contents in whole body. With the further increase in EPBM content in feed, the EE content was decreased, and the serum TG content was increased, which may be caused by peptides that promoted the metabolism of lipids (57).

With the increase in the small peptide contents, the activities of digestive enzymes in the liver and intestine are mostly increasing and then decreasing. It was consistent with the results obtained from sea bass (*Dicentrarchus labrax*) (41), turbot (55), and pacific white shrimp (58). The changing trend of the intestinal digestive enzyme activity was consistent with the VW, VL, and VL/CD value of PI, as well as MT and VL/CD value of DI. The small peptides can accelerate villus growth, promote intestinal digestive function, and induce and stimulate the increase in enzyme activity (59). When FM was replaced by 150 g/kg of EPBM, improvements in digestive enzyme activities and the amelioration of intestinal morphology were important factors in the absence of negative effects on the growth of hybrid grouper.

Active peptides can provide the nitrogen skeleton for digestive enzyme synthesis, promote the secretion of enzymes, and stimulate hormone receptors in the intestine (60). Higher intestinal chymotrypsin and trypsin activities in the EP12 and EP15 groups indicate that increased EPBM required more proteases for adequate protein absorption. This also resulted in higher levels of Alb and TP in serum and CP in the whole body. However, there was no significant action on the composition of large yellow croaker (*Larimichthys crocea*) (42) and turbot (61). The different sources and differences in nutritional ingredients of small peptides are also important reasons for whole-body composition (62).

Antioxidant capacity is commonly referred to as an indicator of aquatic animals’ health (63). Antioxidant enzymes protect against free radical damage, and CAT and SOD can reduce oxidative damage and keep the balance of free radicals (64). A low concentration of reactive oxygen species is one of the necessary conditions for cells to perform normal physiological functions (65). Animals can protect tissues and cells from oxidative stress by regulating CAT and SOD activity (66). Small peptides in feed can improve the antioxidant capacity of hybrid grouper (54), turbot (55), carp (*Carassius auratus gibelio*) (67), and Japanese flounder (*Paralichthys olivaceus*) (68). In this experiment, the changes in CAT and SOD activities were similar to the growth performance, reaching the maximum when FM was replaced with 30 g/kg of EPBM. This suggested that substituting FM with EPBM could improve the antioxidant capacity of the hybrid grouper. The increasement in EPBM treatments for antioxidative ability could be due to the high proportion (472.3 g/kg) of peptides with molecular weight from 500 to 5,000 Da in EPBM. Since previous reports showed that medium-sized bioactive peptides (molecular weight from 300 to 5,000 Da) can have antioxidative and immunity stimulating properties (69). This is also the reason for the increase in ACP activity. The GPT and GOT are important amino acid metabolizing enzymes (70). Under normal circumstances, the aminotransferase activity in serum is low. When animals suffer from various malnutrition, especially when the liver is damaged, serum GOT and GPT activities could increase (71). EPBM replacement of FM reduced the GPT activity in serum, which indicated a protective effect of EPBM on the liver. Similar situations were found on Jian carp (72) and largemouth bass (*Micropterus salmoides*) (73).

The structure and diversity of the intestinal bacterial community directly affect the digestion and absorption, growth performance, and immunity of fish (74). Many factors affect the dynamic balance of the intestinal bacterial community, such as the breeding environment, external stress, and feed composition (75–77). In this study, replacing FM with different levels of EPBM had no significant effect on Chao1 and ACE, indicating no significant effect on species abundance. The Shannon and Simpson indices were not significantly affected by diets, indicating no significant effect on species diversity. There was no variation in the numbers of OTUs, and the number of unique OTUs in each group was 0. The top 10 dominant phylum and dominant genus have no significant difference in category and abundance. These data suggest that the replacement of FM with EPBM may have little effect on the intestinal microbiota of the hybrid grouper. Research has shown that the dominant phylum of vertebrates contains Proteobacteria, Firmicutes, and Bacteroidetes (78). Similar results were obtained in our study, and the dominant phylum of hybrid grouper contained Firmicutes, Bacteroidetes, and Proteobacteria, accounting for 87.07% of the relative abundance. The dominant genus included *uncultured_bacterium_f_Muribaculaceae, Streptococcus*, and *Lactobacillus*, accounting for 31.66% of the relative abundance. These genera of absolute advantage of the intestine in hybrid grouper may be associated with the intestinal microbes that have been set at the seedling stage. Pond et al. believe that after the hatched juvenile fish were exposed to the culture environment, a variety of bacteria will enter the intestinal tract and then set its value in the intestinal epithelium (79). In the future process of growth, the dominant bacterial community was not significantly influenced by the breeding environment and feed.

Amino acid transporter is a functional protein commonly found in the biofilm system (80). It is an important nutrient-sensing molecule and plays a key role in protein synthesis (81). Proteins are absorbed by the body in the form of peptides and amino acids, and the latter is the better absorption form (81), which transport through the peptide transporters (82). The interesting things were that the proper amount of EPBM instead of FM can increase the expression of *LAAT1, CAT2*, and *CAT1* in the PI and DI, *SNAT3* and *B^0^AT2* in the PI, and *B^0, +^AT* in the DI. The relative expression of these genes gradually decreased when the EPBM content of the feed was further increased. This may be attributed to the fact that EPBM is rich in free amino acids. Excess-free amino acids in the feed can lead to saturation of the intestinal amino acid transport system and transport competition, resulting in reduced expression of amino acid transport-related genes, which consequently makes amino acid transport impaired. The proper amount of EPBM instead of FM can increase the expression of *PepT1* in the DI and MI. This might be because EPBM is rich in tripeptides and dipeptides and increased the expression of *PepT1* (83), which allows more small peptide proteins to be absorbed by hybrid grouper. Similar results were obtained on yellow perch (*Perca flavescens*) (84) and rainbow trout (*Oncorhynchus mykiss*) (84). More amino acids were transported from extracellular to intracellular with the increase in amino acid transport expressions (85). The transport of small peptides and amino acids can facilitate each other (86, 87). When hybrid groupers were fed a diet containing 150 g/kg of EPBM, the improved expression of peptide and amino acid transporter was one of the important factors for uninhibited growth performance. In this test, the expression of *PAT1* was not significantly influenced by diet. It may be because *PAT1* is mainly expressed in muscle, and the expression in the intestine is low (88), and the relative change is not obvious.

## Conclusion

This study showed that replacing FM with 30 g/kg of EPBM can significantly improve growth performance, antioxidant capacity, and the ability to transport amino acids and peptides in hybrid grouper.

## Data availability statement

The original contributions presented in this study are included in the article/supplementary material, further inquiries can be directed to the corresponding authors.

## Ethics statement

The animal study was reviewed and approved by the Institutional Animal Care and Use Committee of Guangdong Ocean University. Written informed consent was obtained from the owners for the participation of their animals in this study.

## Author contributions

XY: conceptualization, investigation, formal analysis, and writing – original draft. XZ, GW, XD, QY, HL, and SZ: methodology and resources. BT: methodology, resources, and funding acquisition. SC: conceptualization, writing – reviewing and editing, supervision, project administration, and funding acquisition. All authors have read and approved the final manuscript.

## Conflict of interest

XZ and GW were employed by Yichang Huatai Biological Technology Co., Ltd., Yichang, China. The remaining authors declare that the research was conducted in the absence of any commercial or financial relationships that could be construed as a potential conflict of interest.

## Publisher’s note

All claims expressed in this article are solely those of the authors and do not necessarily represent those of their affiliated organizations, or those of the publisher, the editors and the reviewers. Any product that may be evaluated in this article, or claim that may be made by its manufacturer, is not guaranteed or endorsed by the publisher.
